# Correction: Bright middle cerebellar peduncle sign in multiple system atrophy with predominant cerebellar ataxia is more apparent in double-inversion recovery imaging than in conventional imaging

**DOI:** 10.1371/journal.pone.0315022

**Published:** 2024-12-02

**Authors:** Wataru Shiraishi, Ayano Matsuyoshi, Yusuke Nakazawa, Yukiko Inamori, Akira Yogi, Tetsuya Hashimoto

There are errors in the author affiliations. The correct affiliations are as follows:

Wataru Shiraishi^1,2^, Ayano Matsuyoshi^1,3^, Yusuke Nakazawa^1^, Yukiko Inamori^1^, Akira Yogi^4^, Tetsuya Hashimoto^1,5^

**1** Department of Neurology, Kokura Memorial Hospital, Kitakyushu City, Fukuoka, Japan, **2** Shiraishi Internal Medicine Clinic, Nogata City, Fukuoka, Japan, **3** Department of Neurology, Neurological Institute, Graduate School of Medical Sciences, Kyushu University, Fukuoka, Japan, **4** Department of Radiology, Graduate School of Medical Science, University of the Ryukyus, Nishihara Town, Okinawa, Japan, **5** Department of Comprehensive Strokology, Fujita Health University School of Medicine, Toyoake, Aichi, Japan.
